# IL-1 and its role in rat carrageenan pleurisy

**DOI:** 10.1155/S0962935193000043

**Published:** 1993

**Authors:** R. Goodman, L. R. Mantegna, C. L. McAIlister, E. Bruin, R. L. Dowling, H. George, W. Feeser, B. Freimark, M. Lischwe, S. Pick, R. R. Harris, J. S. Kerr

**Affiliations:** The DuPont Merck Pharmaceutical Company Inflammatory Diseases Research Experimental Station E400/4223 Wilmington DE 19898-0400 USA

## Abstract

The carrageenan pleurisy model, which is characterized by cellular influx and oedema, has been used to examine the effects of anti-inflammatory compounds such as naproxen. Interleukin-1α and β (IL-1) are known to be pro-inflammatory mediators, and their roles in this model are unknown. Intrapleural injection of 1% viscarin carrageenan or saline was administered to male Lewis rats. Four to 24 h later, cell counts, fluid volumes and IL-1β levels (measured by ELISA) were determined in the pleural cavity. Serum corticosterone levels were measured only at 4 h. Significant increases in IL-1β levels precede cell influx suggesting IL-1β plays a role in the maintenance of cell accumulation in the pleural cavity. None of the drugs tested, including the IL-1 receptor antagonist, maintained pleural cell influx and IL-1β levels at control levels. When human IL-1α or β or rat IL-1β were injected individually into the pleural cavity, none of these cytokines were pro-inflammatory, as measured by increased cell influx and fluid extravasation. These results suggest that although IL-1β levels increase in the pleural cavity in response to carrageenan, IL-1 per se is not the initiator of the pro-inflammatory events of cell influx and oedema in this model.


Mediators of Inflammation, 2, 33–39 (1993)

### IL-1 and its role in rat carrageenan pleurisy

R. Goodman, L. R. Mantegna, C. L. McAllister, E. Bruin, R. L. Dowling, H. George, W. Feeser, B. Freimark, M. Lischwe, S. Pick, R. R. Harris and J. S. Kerr[CA]

The DuPont Merck Pharmaceutical Company, Inflammatory Diseases Research, Experimental Station E400/4223, Wilmington, DE 19898-0400, USA

[CA]Corresponding Author

**THE carrageenan pleurisy model, which is characterized by cellular influx and oedema, has been used to examine the effects of anti-inflammatory compounds such as naproxen. Interleukin-1α and β (IL-1) are known to be pro-inflammatory mediators, and their roles in this model are unknown. Intrapleural injection of 1% viscarin carrageenan or saline was administered to male Lewis rats. Four to 24 h later, cell counts, fluid volumes and IL-1β levels (measured by ELISA) were determined in the pleural cavity. Serum corticosterone levels were measured only at 4 h. Significant increases in IL-1β levels precede cell influx suggesting IL-1β plays a role in the maintenance of cell accumulation in the pleural cavity. None of the drugs tested, including the IL-1 receptor antagonist, maintained pleural cell influx and IL-1β levels at control levels. When human IL-1α or β or rat IL-1β were injected individually into the pleural cavity, none of these cytokines were pro-inflammatory, as measured by increased cell influx and fluid extravasation. These results suggest that although IL-1β levels increase in the pleural cavity in response to carrageenan, IL-1 *per se* is not the initiator of the pro-inflammatory events of cell influx and oedema in this model.**

**Key words:** Carrageenan, Cytokines, IL-1, Inflammation, Pleurisy

### Introduction

The components of inflammation include leukocyte infiltration and fluid accumulation which accompany the cardinal signs of inflammation such as heat, swelling, redness and pain. Many mediators, including lipids, proteinases, biogenic amines, and peptides have been implicated in this process. In order to investigate the roles of such mediators, many animal models have been developed. One such model is carrageenan induced pleurisy in the rat, in which the phlogistic agent is usually viscarin carrageenan. This model has been extensively studied, has the advantage of being versatile, and has been used to evaluate standard anti-inflammatory agents.[1–5]

Using the carrageenan pleurisy model the potential involvement of interleukin-1(IL-1), a cytokine which is a known pro-inflammagen,[6] has been investigated. Among its many activities IL-1 can stimulate cells to induce synthesis of other cytokines,[7] increase activity of enzymes such as phospholipase A2,[8,9] and is responsible for the accumulation of neutrophils at the sites of inflammation.[10] The exact role of IL-1 in the inflammatory process induced by carrageenan is still unclear. The purpose of this study was to investigate the role of IL-1 and the IL-1 receptor antagonist in this model.

### Materials and Methods

*Reagents:* Human and rat IL-1 α and β, human IL-1 receptor antagonist (rhIL-1ra), and rabbit polyclonal anti-mouse IL-1 antibodies were prepared at The Du Pont Merck Pharmaceutical Co. All other reagents were purchased from Sigma Chemical Company, St. Louis, MO, or through VWR, and were reagent grade.

*Pleurisy:* Male rats (Lewis rats, LEW/Crl BR Charles River Laboratories, Wilmington, MA) weighing 200–250 g were divided into groups of ten. The procedures have been described previously.[1–4] Pleurisy tests were carried out using either rats fasted for 16 h prior to the study or rats fed *ad libitum*. Animals were treated orally with appropriate drugs or subcutaneously with the rhIL-1ra 1 h prior to the introduction of the inflammagen. Animals were anaesthetized with ether and placed left side down and injected intrapleurally with 0.2 ml of 1% viscarin carrageenan (Sigma Chemical Co.) or rat IL-1β, human IL-1α or β at doses described. Four to 24 h after injection animals were euthanized using $CO_2$. An intercostal incision was made in the pleural cavity, the cavity lavaged with 6 ml of 0.9% NaCl, exudate volumes recorded, and fluids centrifuged at 658 × ***g*** (Sorvall RC5C, Du Pont, Newtown, CT) for 10 min at 5°C. Samples

© 1993 Rapid Communications of Oxford Ltd overtly bloody were discarded. A 1 ml sample of supernatant was saved and stored at 4°C for determination of IL-1$\beta$ levels by ELISA. The remaining supernatant was decanted and cells resuspended in 2 ml of 0.9% NaCl containing 2% heparin. Aliquots were used to determine total cell (Coulter Counter, Coulter Corp, Hialeah, FL) and differential cell counts using Wright's stain. Changes in fluid volumes were calculated as the difference between the initial 6 ml used for the lavage and the final volume recovered from the pleural cavity.

*ELISA:* A sandwich ELISA,[11] using a polyclonal rabbit antiserum obtained by immunization with murine recombinant IL-1$\beta$, was used to measure the IL-1$\beta$ levels from the rat pleural cavities. The microtitre wells were coated with a protein A purified rabbit-anti-mIL-1$\beta$ IgG (20 $\mu$g/ml in phosphate buffered saline) at room temperature for 1 h. The wells were washed with Dulbecco's phosphate buffered saline. Volumes (100 $\mu$l) of sample or known recombinant IL-1$\beta$ dilutions were added to the wells, followed by a 2 h incubation at room temperature. The washing step was repeated, and 100 $\mu$l of the biotinylated affinity-purified rabbit-anti-mIL-1$\beta$ antibody was added and allowed to incubate at room temperature for 1 h. The optimal dilution of this secondary antibody was determined by titre prior to the assay. The assay was developed using avidin coupled to alkaline phosphatase followed by *p*-nitrophenyl phosphate substrate. Incubation was continued at room temperature for 90 min, and the plates were read at 405 nm (UV Kinetics microplate reader, Molecular Devices Corp., Menlo Park, CA). The concentration of IL-1$\beta$ in the samples was calculated from the IL-1$\beta$ standard curve. This assay was specific for IL-1$\beta$; IL-1$\alpha$ and the rhIL-1ra did not cross-react. The lower level of detection for IL-1$\beta$ was 0.1 ng/ml.

*Recombinant human and rat IL-1$\alpha$ and $\beta$ and recombinant human IL-1 receptor antagonist:* All IL-1 proteins[12] and the rhIL-1ra were produced in *Escherichia coli* and purified by standard column chromatography methods. Samples were tested for endotoxin using the LAL assay (Whittaker M.A. Bioproducts) with all preparations shown to contain less than 10 EU/mg of protein. The characteristics of the rhIL-1ra were identical to those published previously.[13]

*Corticosterone levels:* Blood was collected into vacutainers containing sodium heparin from anaesthetized animals via heart puncture. Plasma samples were obtained by centrifugation for 10 min at 658 × ***g***. Samples were assayed using an RIA for corticosterone, the major glucocorticoid in rat plasma (MetPath Inc., Rockville, MD). The normal range for this assay is 10–60 ng/ml.[14]

*Statistical analysis:* Data are expressed as mean ± standard error of the mean, determined by analysis of variance followed by Duncan's multiple range test (SAS Institute, Cary, NC). Statistical significance was considered to be $p < 0.05$.

### Results

*Time course:* Figure 1A shows the response to carrageenan over a 24 h time course. Cell influx increased 13-fold from $1 \times 10^7$ cells/ml (30 min) to $1.3 \times 10^8$ cells/ml (4 h) and remained elevated through 24 h ($1.4 \times 10^8$ cells/ml) ($p < 0.05$). At 4 h the cells present were >90% PMN's and by 24 h > 50% mononuclear cells were found. These percentages are consistent with previous findings.[2] Fluid extravasation significantly increased from 0.2 ml in saline treated animals to a maximum of 2.5 ml in carrageenan treated animals after 16 h ($p < 0.05$). Cell influx remained constant through 24 h, while fluid volume changes significantly decreased from 16 through 24 h ($p < 0.05$).

Figure 1B shows IL-1$\beta$ levels in response to carrageenan. IL-$\beta$ increased 2.4-fold from 0.3 ng/ml (30 min) to 0.72 ng/ml (1 h), and remained elevated through 6 h (0.54 ng/ml), returning to background by 16 h (0.15 ng/ml) ($p < 0.05$). The changes in the IL-1$\beta$ levels in the pleural cavity occurred quickly, and preceded the changes in cell influx or fluid volumes.

*Drugs:* The anti-inflammatory drugs dexamethasone, piroxicam, and naproxen were tested in this model at 4 h, a time at which cells, fluid volume and IL-1$\beta$ levels were significantly elevated in the carrageenan treated rats. None of these drugs maintained cell influx and fluid volumes at control levels. At all doses tested, cell influx was approximately midway between the saline and the carrageenan treated animals (Fig. 2A). (Dexamethasone was dosed from 0.01–10 mg/kg; piroxicam was dosed from 1–10 mg/kg; naproxen was dosed at 40 and 100 mg/kg.) Fluid extravasation was significantly reduced in all drug doses by 15%, which was equivalent to 0.7 ml (data not shown).

IL-1$\beta$ levels decreased in the drug treated rats, but not in a dose dependent manner. Dexamethasone decreased IL-1$\beta$ levels by 64%, from 0.62 ng/ml at 0.03 mg/kg to 0.21 ng/ml at 1 mg/kg ($p < 0.05$). Piroxicam did not significantly reduce IL-1$\beta$ levels (0.51 ng/ml). Naproxen, tested at a single dose of 40 mg/kg, reduced IL-1$\beta$ levels by 35%, from 0.51 ng/ml (controls) to 0.33 ng/ml at 40 mg/kg (Fig. 2B).

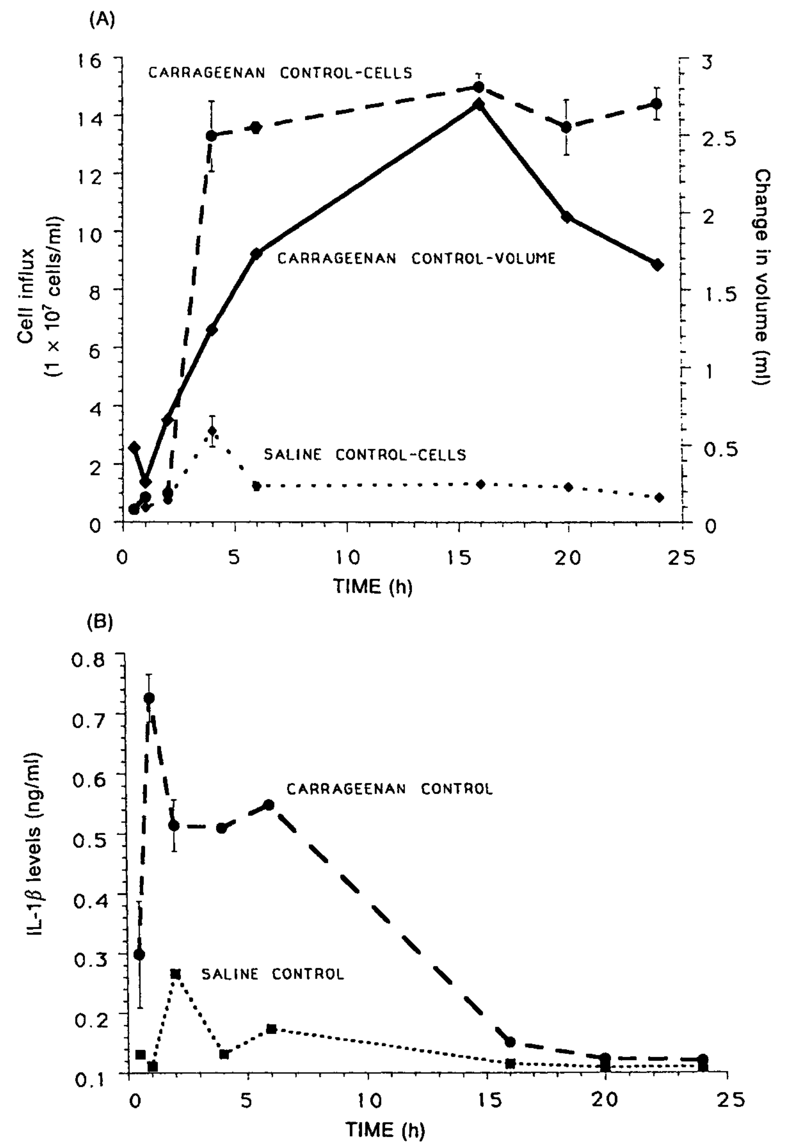

FIG. 1 (A) Time course of cell influx and fluid extravasation in the pleural cavity. Cell influx was measured in both saline treated (—◆—) and carrageenan treated (—●—) animals. Fluid volume changes in carrageenan treated animals (-◆-) is shown with no differences at any time with saline treated control (data not shown). Each time point represents mean ± S.E.M.; $n = 4$. Curves are significantly different from 2–24 h for both cell influx and change in volume for carrageenan treated animals compared with saline treated controls ($p < 0.05$). (B) Time course of IL-1β levels in the pleural cavity with carrageenan treated (—●—) or saline treated (-■-) animals. Each time point represents mean ± S.E.M.; $n = 4$. Curves are significantly different from 0.5–10 h ($p < 0.05$).

*rhIL-1ra:* The rhIL-1ra also maintained cell influx approximately midway between the saline and carrageenan controls (Fig. 2A). The maximum reduction in cell influx was observed at the 1 mg/kg dose, decreasing the cells from $9.5 \times 10^7$ cells/ml to $7.3 \times 10^7$ cells/ml (23%; NS). The rhIL-1ra significantly ($p < 0.05$) increased IL-1β levels from 0.50 ng/ml to 0.78 ng/ml at 40 mg/kg in a non-dose dependent manner (Fig. 2B).

*Fed* versus *fasted rats:* Fasting is a form of stress, and both plasma corticosterone and IL-1 levels may increase with stress.[14] The effect of fasting on total cell counts, fluid extravasation and IL-1β levels in the pleural cavity, and plasma corticosterone levels was investigated. The saline treated fed or fasted animals each had a total cell count of $1.5–1.7 \times 10^7$ cells/ml. We compared total cell counts, fluid extravasation and IL-1β levels in rats fed *ad libitum* and rats fasted for 16 h dosed with rhIL-1ra. In fed animals total cell counts ranged from $1.2 \pm 0.3 \times 10^8$ cells/ml (carrageenan treated) to $7 \pm 0.5 \times 10^7$ cells/ml with animals dosed with 80 mg/kg rhIL-1ra ($p < 0.05$). In fasted animals total cell counts ranged from $1.0 \pm 0.03 \times 10^8$ cells/ml (carrageenan treated) to $7.0 \pm 1.3 \times 10^7$ cells/ml in animals dosed with 10 mg/kg rhIL-ra ($p < 0.05$; Fig. 3). There were no significant increases in

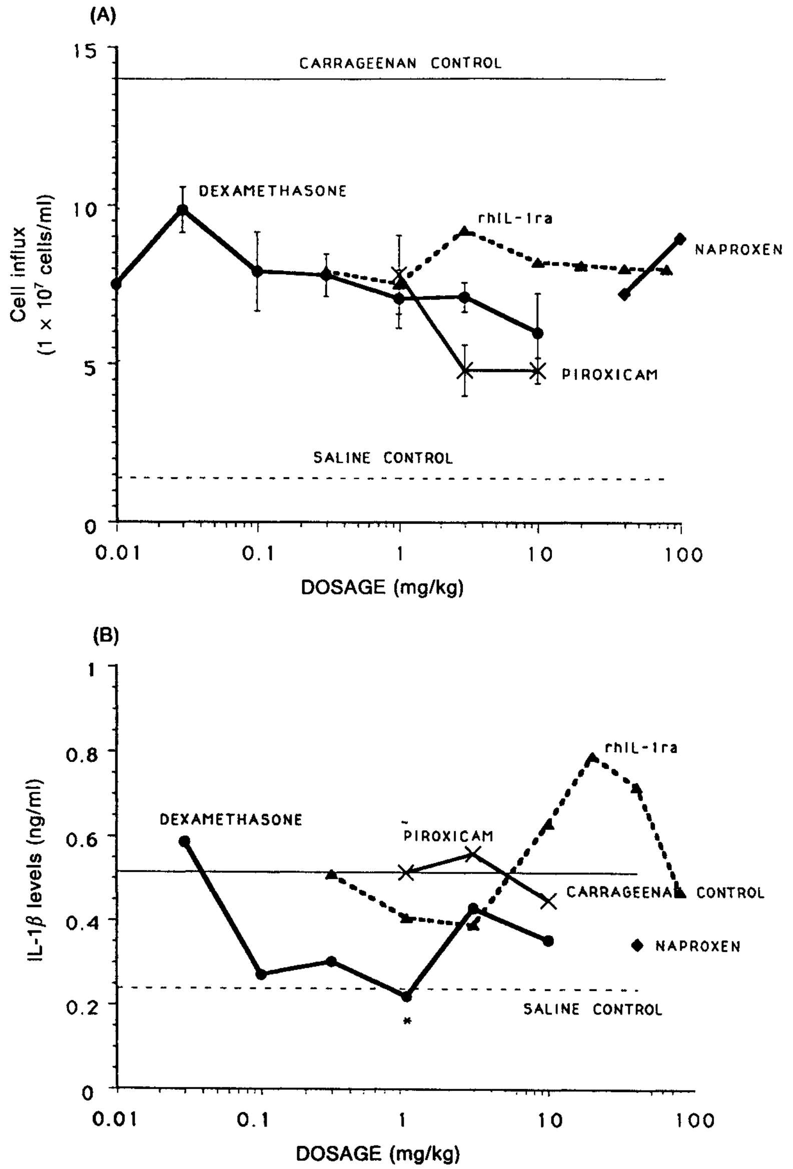

FIG. 2(A) Effect of dexamethasone (–●–), naproxen (–◆–), piroxicam (–×–) and the rhIL-1ra (–▲–) on cell influx in the pleural cavity. Each dose represents mean ± S.E.M.; $n$ = 4–11. All curves are significantly different from both the carrageenan and saline groups ($p < 0.05$). (B) Effect of dexamethasone, naproxen, piroxicam and the rhIL-1ra on IL-1$\beta$ levels in the pleural cavity. Each dose represents mean ± S.E.M.; $n$ = 4–11. * Compared with the carrageenan control ($p < 0.05$).

fluid volumes with any of the dosing regimens in the carrageenan treated fasted or fed rats compared with saline treated controls (data not shown). IL-1$\beta$ levels in lavaged pleural cavities from fed animals dosed with rhIL-1ra were significantly increased compared with saline controls, returning toward controls with 80 mg/kg (Table 1). Fasted animals had IL-1$\beta$ levels ranging from 0.40 ± 0.04 ng/ml (carrageenan treated animals) to 0.79 ± 0.14 ng/ml in animals dosed with 20 mg/kg rhIL-1ra. All IL-1$\beta$ levels were significantly increased compared with saline controls (0.25 ± 0.01 ng/ml) (Table 1).

*Corticosterone levels:* Plasma levels were measured in the fed and fasted rats as an indication of stress (Table 2). Normal rat corticosterone levels range from 10 to 60 ng/ml.[14] The rhIL-1ra had no effect on plasma corticosterone levels in fasted rats. The fed carrageenan treated animals had corticosterone levels of 485 ± 21 ng/ml. In fed animals, as the doses of rhIL-1ra increased from 3 to 80 mg/kg, corticosterone levels decreased from 502 ± 35 ng/ml to 42 ± 7 ng/ml in a dose dependent manner (Table 1; $p < 0.05$), returning to the normal range. In comparison, in fasted animals corticosterone levels did not decrease in a dose dependent manner (Table 2); nor did the corticosterone levels return to the normal range (10–60 ng/ml), even at 80 mg/kg. At 40 and 80 mg/kg rhIL-1ra, the corticosterone levels were significantly increased in the fasted animals compared with the fed ones. These data demonstrate that carrageenan admin-

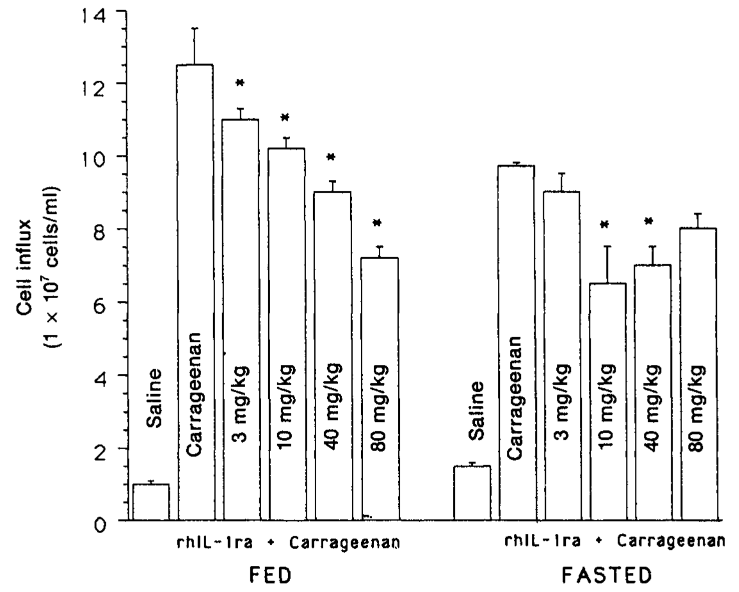

FIG. 3. Total cells lavagable from the pleural cavity of rats fed *ad libitum* or fasted for 16 h prior to carrageenan injection with or without rhIL-1ra. $n = 6$. * Compared with the carrageenan controls ($p < 0.05$).

istration significantly increases plasma corticosterone and the stress of fasting increases the variability of the plasma corticosterone levels.

*Intrapleural injection of human IL-1β* or α or rat IL-1β: IL-1β (human and rat) and IL-1α (human) were injected intrapleurally to determine if any of these cytokines are pro-inflammagens by themselves. There were no significant changes in cell influx or fluid volumes from 30 min to 24 h with any of the cytokines tested in doses ranging from 0.001–100 μg/rat for human and rat IL-1β and 50–200 μg/rat for human IL-1α (Fig. 4).

### Discussion

Carrageenan is an irritant which sets up a cascade of events that leads to cell and fluid influx in the pleural cavity, as described previously.[1-5] Small blood vessels in the subpleural tissues become more permeable, allowing serum proteins, proteinases such as complement, and cells to move into the pleural cavity. The serum complement components may be responsible for the cell influx, since many are chemotactic. Complement inhibitors are active in this model within the first hour after the administration of the carrageenan.[1] The purpose of the present study was to further characterize the model by examining the possible role of IL-1.

The total number of cells recoverable from the pleural cavity at the end of 4 h was $1.2 \times 10^8$ cells/ml, which is consistent with the cell influx reported 6 h after carrageenan administration.[2] IL-1β levels were maximum at 1 h, preceding the maximal influx of cells. However, cells remained elevated for 24 h, by which time IL-1 levels had returned to control. This observation suggests that IL-1 may play a role in attracting cells into the pleural cavity.

Since IL-1 was present in the pleural cavity, the possibility that the IL-1 receptor antagonist affected

**Table 1.** IL-1β levels in male Lewis rats fasted 16 h or fed *ad libitum*

| Treatment | | IL-1β Levels (ng/ml) | |
|---|---|---|---|
| | | Fasted | Fed |
| Carrageenan control | | 0.40 ± 0.04* | 0.29 ± 0.03* |
| Saline control | | 0.25 ± 0.01 | 0.18 ± 0.01 |
| Carrageenan + rhIL-1ra | 3.0 | 0.52 ± 0.03* | 0.27 ± 0.03* |
| (mg/kg) | 10.0 | 0.68 ± 0.07* | 0.31 ± 0.03* |
| | 20.0 | 0.79 ± 0.14* | 0.32 ± 0.03* |
| | 40.0 | 0.39 ± 0.01* | 0.38 ± 0.04* |
| | 80.0 | 0.40 ± 0.04* | 0.24 ± 0.03 |

*$p < 0.05$ compared with saline controls.
$n = 5$.

**Table 2.** Plasma corticosterone levels in male Lewis rats

| Treatment | | Corticosterone levels (ng/ml) | |
|---|---|---|---|
| | | Fasted | Fed |
| Carrageenan control | | 415 ± 46* | 485 ± 21* |
| Saline control | | 168 ± 68 | 290 ± 72 |
| Carrageenan + rhIL-1ra | 3.0 | 391 ± 39* | 502 ± 35* |
| (mg/kg) | 10.0 | 331 ± 55* | 317 ± 35* |
| | 20.0 | 177 ± 34 | 129 ± 43 |
| | 40.0 | 321 ± 118* | 57 ± 11† |
| | 80.0 | 139 ± 19 | 42 ± 7† |

*$p < 0.05$ compared with saline control.
$n = 4$.
† $p < 0.05$ compared with fasted group.
Normal corticosterone levels 10–60 ng/ml.

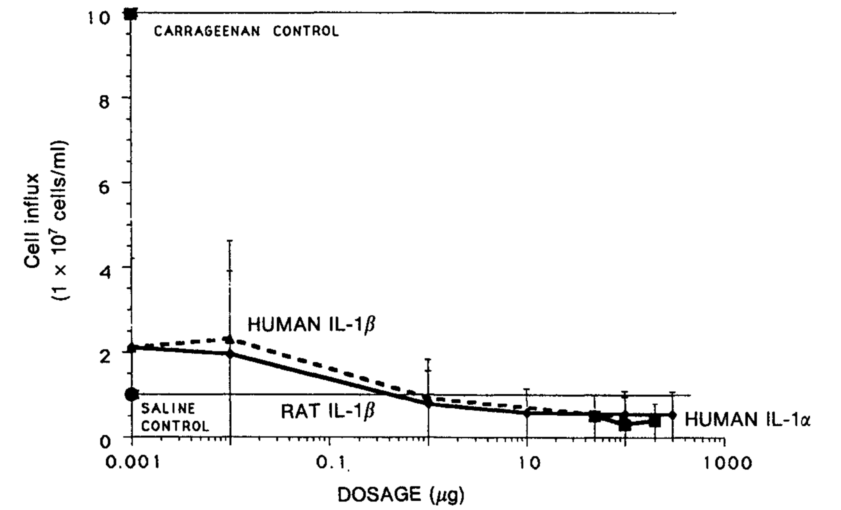

FIG. 4. Cell counts recoverable from the pleural cavity of rats injected with rat IL-1β (–●–), human IL-1β (–▲–) or human IL-1α (–■–). There are no significant differences from the saline treated controls with any protein at any concentration, $n = 3$.

cell influx, fluid extravasation and IL-1β levels was investigated. Since the standard drugs dexamethasone and naproxen reduce cell and fluid influx in this model,[2,16] these were administered for comparison. Cell influx in all the drug treated rats was significantly increased compared with the saline controls ($1.5 \times 10^6$ cells/ml) and significantly less than the level of the carrageenan controls ($1.45 \times 10^8$ cells/ml). The effects of dexamethasone and naproxan are consistent with previous findings. The $ED_{30}$ values for cellular infiltration with orally administered dexamethasone is 0.05 mg/kg, naproxen is 1.4 mg/kg and piroxicam is 0.7 mg/kg, indicating that these drugs are active in this model.[15] Fluid volumes significantly decreased by approximately 15% with IL-1β levels remaining constant. These data substantiate the involvement of several mechanisms in the inflammatory process induced by carrageenan, since individually none of the drugs prevented the pro-inflammatory changes in the pleural cavity.

The IL-1 receptor antagonist inhibits IL-1 by binding to its receptors and has no apparent agonist activity.[13] It has been shown to be active in several septic shock models,[16,17] increasing animal survival. Administration of this protein does not completely prevent IL-1β influx or cell accumulation in the pleural cavity in response to carrageenan. Meyers *et al.*[18] have reported that in rats fed *ad libitum*, the rhIL-1ra decreased total cell influx in a dose dependent manner from 28% to 74% at doses ranging from 0.3–10 mg/kg. In our model using rats fed *ad libitum*, the rhIL-1ra did decrease cell influx, but by no more than 45%. The response in the fasted animals was shown to be more variable, not dose dependent and the maximum inhibition of cell influx was approximately 35%. In general, the IL-1β levels in the pleural cavity were higher in the fasted animals than in the fed ones. Therefore, fasting and carrageenan induced inflammation together lead to a response that requires more rhIL-1ra to down-regulate it.

IL-1 is one of the key mediators of the brain–endocrine immune response to stress.[19] IL-1 activates the hypothalamic–pituitary–adrenal axis; its effect on this axis appears to be mediated by activation of secretion of corticotropin releasing factor, leading to the release of ACTH and corticosterone.[20,21] In stress there are elevations in plasma corticosterone and IL-1 levels in rats.[14] We stressed our animals by fasting them for 16 h prior to administration of the carrageenan. We hypothesized that this stress should increase plasma corticosterone levels and may increase IL-1 levels in the pleural cavity resulting in higher doses of the rhIL-1ra to down-regulate this response. The rhIL-1ra should lead to a reduction in the IL-1 induction of plasma corticosterone levels. Carrageenan does induce increased plasma corticosterone levels compared with controls whether the animals are fed or fasted. We observed more variability in the response of fasted animals to the rhIL-1ra. In the rats fed *ad libitum*, plasma corticosterone levels decreased in a dose dependent manner as the dose of the rhIL-1ra increased, suggesting that IL-1-induced corticosterone secretion was effectively inhibited. Corticosterone levels in the food deprived animals did not return to normal. IL-1 levels in the pleural cavity only returned toward the saline controls at the highest dose of the receptor antagonist. These findings demonstrate that 16 h of food deprivation does play a role in the response of the animal to carrageenan, both in its plasma corticosterone and pleural cavity IL-1$\beta$ levels, and suggest that IL-1 may mediate this response.

In order to investigate the role of IL-1 as a pro-inflammatory agent in the pleurisy model, we injected human and rat IL-1$\beta$ and human IL-1$\alpha$ directly into the pleural cavity. When the human and rat IL-1$\alpha$ and $\beta$ were injected across a wide range, there was no inflammatory response in the pleural cavity, as determined by changes in cell influx or fluid volume. These data suggest that IL-1 is an important component in the amplification of inflammation, but that it is not the initiator of the inflammatory events. In this model several inflammatory mediators including histamine, bradykinin and thromboxane have been detected in the pleural exudates of rats.[1] The role these mediators play in exudate formation is unclear, since the injection of any one of these mediators alone does not result in a significant fluid or cellular response.[1] Thus, the observation that IL-1 *per se* is not a pro-inflammagen is consistent with the findings for other mediators. The combination of one or more of such mediators may be responsible for the inflammation.

The present work demonstrates that levels of IL-1$\beta$ are found in the pleural cavity of the carrageenan injected rats in association with increased cellular influx and oedema. The stress of 16 h of food deprivation also influences the response of the animals to the carrageenan induced injury, indicated by increased plasma corticosterone and pleural IL-1$\beta$ levels. However, human IL-1$\alpha$ and $\beta$ and rat IL-1$\beta$ are not pro-inflammatory agents when injected individually into the rat pleural cavity under the conditions we used, suggesting that IL-1 *per se* is not the initiator of cell influx and fluid extravasation in this model.

### References

1. De Brito FB. Pleurisy and pouch models of acute inflammation. In: Chang JY, Lewis AJ, eds. *Pharmacological Methods in the Control of Inflammation.* NY: Alan R Liss, Inc. 1989; 173–194.
2. Ackerman N, Tomolonis A, Miram L, Kheifets J, Martinez S, Carter A. Three day pleural inflammation: A new model to detect drug effects on macrophage accumulation. *J Pharm Exp Ther* 1980; **215**: 588–595.
3. Vinegar R, Truax JF, Selph JL, Voelker FA. Pathway of onset, development, and decay of carrageenan pleurisy in the rat. *Fed Proc* 1982; **41**: 2588–2595.
4. Bliven ML, Otterness IG. Carrageenan pleurisy. In: G. DiSabato, ed. *Methods in Enzymology.* NY: Academic Press 1988; **162**: 334–339.
5. Utsunomiya I, Nagai S, Oh-Ishi S. Sequential appearance of IL-1 and IL-6 activities in rat carrageenan-induced pleurisy. *J Immun* 1991; **147**: 1803–1809.
6. Dinarello CD. Biology of interleukin 1. *FASEB J* 1988; **2**: 108–115.
7. Le J, Vilcek J. Interleukin 6: A multifunctional cytokine regulating immune reactions and the acute phase protein response. *Lab Invest* 1989; **61**: 588–602.
8. Kerr JS, Stevens TM, Davis GL, McLaughlin JA, Harris RA. Effects of recombinant interleukin-1 beta on phospholipase A2 activity, phospholipase A2 mRNA levels, and eicosanoid formation in rabbit chondrocytes. *Biochem Biophys Res Commun* 1989; **165**: 1079–1084.
9. Goodman R, Stevens TM, Mantegna RL, Kidd PR, Harris RR, Kerr JS. *Phospholipase A2 (PLA2) activity in bovine pulmonary artery endothelial cells. Agents & Actions* 1991; **34**: 113–116.
10. Beck G, Habicht GS, Benach JL, Miller F. Interleukin 1: A common endogenous mediator of inflammation and local Shwartzman reaction. *J Immunol* 1986; **136**: 3025–3031.
11. McMaster MT, Newton RC, Dey SK, Andrews GK. Activation and distribution of inflammatory cells in the mouse uterus during the preimplantation period. *J Immunol* 1992; **148**: 1699–1705.
12. Huang JJ, Newton RC, Pezzella K, *et al.* High-level expression in *Escherichia coli* of a soluble and fully active recombinant interleukin-1$\beta$. *Mol Biol Med* 1987; **4**: 169–181.
13. Eisenberg SP, Evans RJ, Arend WP, *et al.* Primary structure and functional expression from complementary cDNA of a human IL-1 receptor antagonist. *Nature* 1990; **343**: 341–346.
14. Meeks RG. The rat. In: Loeb W, Quimby FW, eds. *The Clinical Chemistry of Laboratory Animals.* NY: Pergamon Press, 1989; 19–25.
15. Berkenkopf JW, Weichman BM. Differential effects of anti-inflammatory drugs on fluid accumulation and cellular infiltration in reverse passive Arthus pleurisy and carrageenan pleurisy in rats. *Pharmacology* 1987; **34**: 309–325.
16. Alexander HR, Doherty GM, Buresh CM, Venzon DJ, Norton JA. A recombinant human receptor antagonist to interleukin 1 improves survival after lethal endotoxemia in mice. *J Exp Med* 1991; **173**: 1029–1031.
17. Fischer E, Marano MA, Van Zee KJ, *et al.* Interleukin-1 receptor blockade improves survival and hemodynamic performance in *Escherichia coli* septic shock, but fails to alter host responses to sublethal endotoxemia. *J Clin Invest* 1992; **89**: 1551–1557.
18. Meyers K, Czachowski R, Hageman R, Thompson RC, Welton AF, Coffey JW. Effect of treatment with recombinant IL-1 receptor antagonist (IL-1ra) on the development of carrageenan-induced pleurisy. *FASEB J* 1990; **4**: A1767.
19. De Souza EB, Webster EL, Grigoriadis DE, Tracey DE. Corticotropin-releasing factor (CRF) and interleukin-1 (IL-1) receptors in the brain–pituitary–immune axis. *Psychol Bull* 1989; **25**: 299–305.
20. Cambronero JC, Rivas FJ, Borrell J, Guaza C. Interleukin-1-beta induces pituitary adrenocorticotropin secretion: Evidence for glucocorticoid modulation. *Neuroendrinology* 1992: **55**: 648–654.
21. Hermus ARMM, Sweep CGJ. Cytokines and the hypothalamic-pituitary adrenal axis. *J Steroid Biochem Mol Biol* 1990; **37**: 867–871.

**Received 16 September 1992; accepted in revised form 24 November 1992.**
